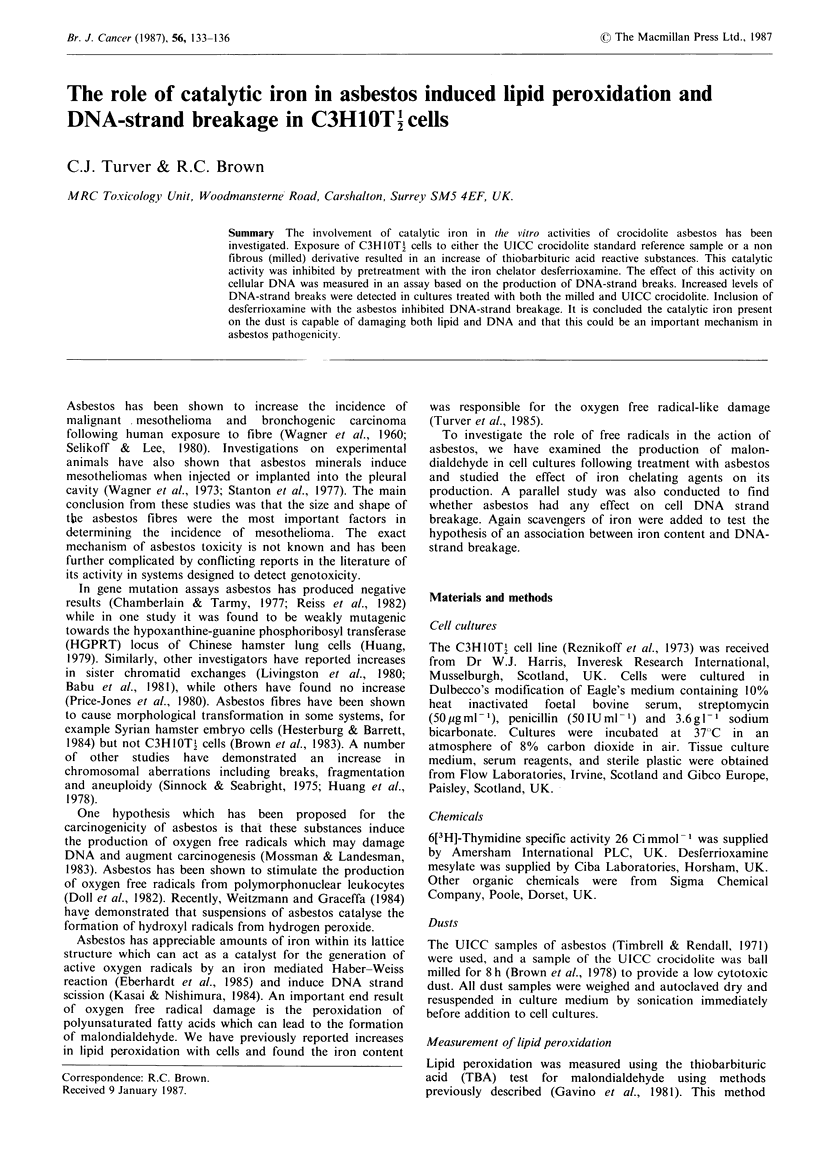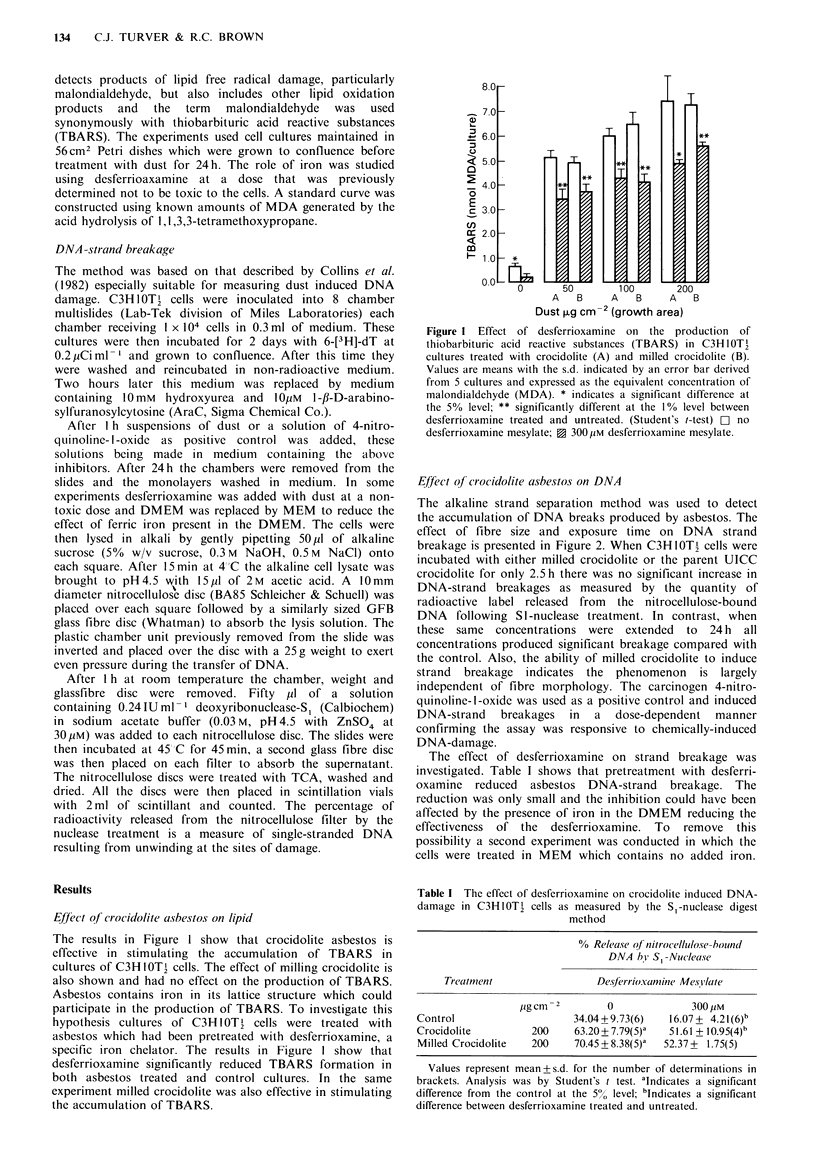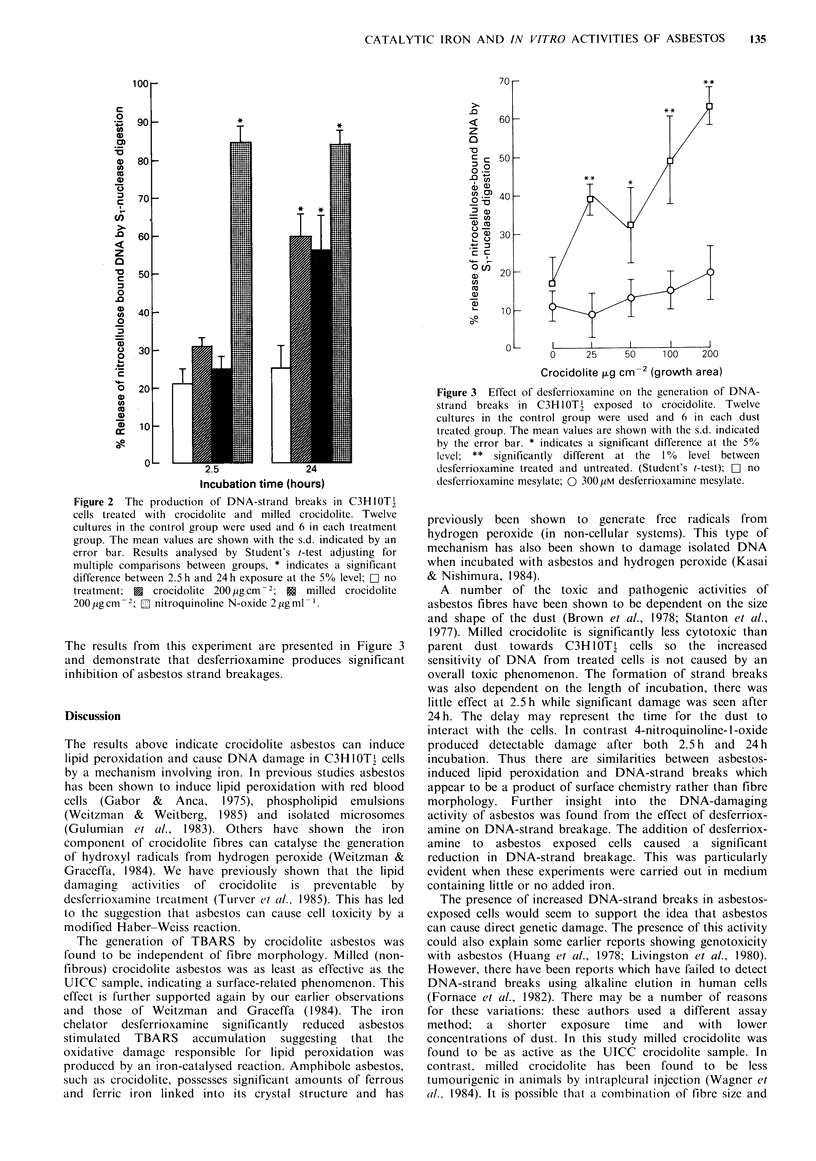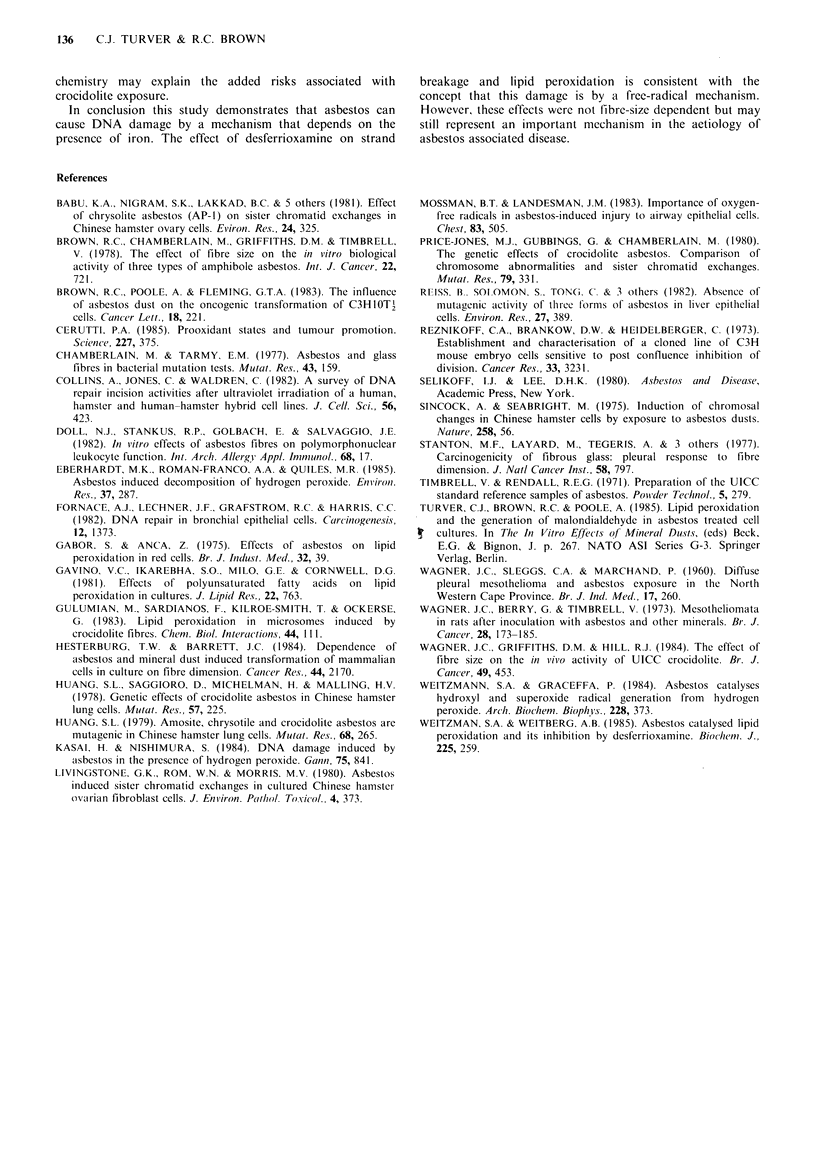# The role of catalytic iron in asbestos induced lipid peroxidation and DNA-strand breakage in C3H10T1/2 cells.

**DOI:** 10.1038/bjc.1987.170

**Published:** 1987-08

**Authors:** C. J. Turver, R. C. Brown

**Affiliations:** MRC Toxicology Unit, Carshalton, Surrey, UK.

## Abstract

The involvement of catalytic iron in the vitro activities of crocidolite asbestos has been investigated. Exposure of C3H10T1/2 cells to either the UICC crocidolite standard reference sample or a non fibrous (milled) derivative resulted in an increase of thiobarbituric acid reactive substances. This catalytic activity was inhibited by pretreatment with the iron chelator desferrioxamine. The effect of this activity on cellular DNA was measured in an assay based on the production of DNA-strand breaks. Increased levels of DNA-strand breaks were detected in cultures treated with both the milled and UICC crocidolite. Inclusion of desferrioxamine with the asbestos inhibited DNA-strand breakage. It is concluded the catalytic iron present on the dust is capable of damaging both lipid and DNA and that this could be an important mechanism in asbestos pathogenicity.


Br. J. Cancer (1987), 56, 133–136 © The Macmillan Press Ltd., 1987

### The role of catalytic iron in asbestos induced lipid peroxidation and DNA-strand breakage in C3H10T½ cells

C.J. Turver & R.C. Brown

*MRC Toxicology Unit, Woodmansterne Road, Carshalton, Surrey SM5 4EF, UK.*

**Summary** The involvement of catalytic iron in *the vitro* activities of crocidolite asbestos has been investigated. Exposure of C3H10T½ cells to either the UICC crocidolite standard reference sample or a non fibrous (milled) derivative resulted in an increase of thiobarbituric acid reactive substances. This catalytic activity was inhibited by pretreatment with the iron chelator desferrioxamine. The effect of this activity on cellular DNA was measured in an assay based on the production of DNA-strand breaks. Increased levels of DNA-strand breaks were detected in cultures treated with both the milled and UICC crocidolite. Inclusion of desferrioxamine with the asbestos inhibited DNA-strand breakage. It is concluded the catalytic iron present on the dust is capable of damaging both lipid and DNA and that this could be an important mechanism in asbestos pathogenicity.

Asbestos has been shown to increase the incidence of malignant mesothelioma and bronchogenic carcinoma following human exposure to fibre (Wagner *et al.*, 1960; Selikoff & Lee, 1980). Investigations on experimental animals have also shown that asbestos minerals induce mesotheliomas when injected or implanted into the pleural cavity (Wagner *et al.*, 1973; Stanton *et al.*, 1977). The main conclusion from these studies was that the size and shape of the asbestos fibres were the most important factors in determining the incidence of mesothelioma. The exact mechanism of asbestos toxicity is not known and has been further complicated by conflicting reports in the literature of its activity in systems designed to detect genotoxicity.

In gene mutation assays asbestos has produced negative results (Chamberlain & Tarmy, 1977; Reiss *et al.*, 1982) while in one study it was found to be weakly mutagenic towards the hypoxanthine-guanine phosphoribosyl transferase (HGPRT) locus of Chinese hamster lung cells (Huang, 1979). Similarly, other investigators have reported increases in sister chromatid exchanges (Livingston *et al.*, 1980; Babu *et al.*, 1981), while others have found no increase (Price-Jones *et al.*, 1980). Asbestos fibres have been shown to cause morphological transformation in some systems, for example Syrian hamster embryo cells (Hesterburg & Barrett, 1984) but not C3H10T½ cells (Brown *et al.*, 1983). A number of other studies have demonstrated an increase in chromosomal aberrations including breaks, fragmentation and aneuploidy (Sinnock & Seabright, 1975; Huang *et al.*, 1978).

One hypothesis which has been proposed for the carcinogenicity of asbestos is that these substances induce the production of oxygen free radicals which may damage DNA and augment carcinogenesis (Mossman & Landesman, 1983). Asbestos has been shown to stimulate the production of oxygen free radicals from polymorphonuclear leukocytes (Doll *et al.*, 1982). Recently, Weitzmann and Graceffa (1984) have demonstrated that suspensions of asbestos catalyse the formation of hydroxyl radicals from hydrogen peroxide.

Asbestos has appreciable amounts of iron within its lattice structure which can act as a catalyst for the generation of active oxygen radicals by an iron mediated Haber–Weiss reaction (Eberhardt *et al.*, 1985) and induce DNA strand scission (Kasai & Nishimura, 1984). An important end result of oxygen free radical damage is the peroxidation of polyunsaturated fatty acids which can lead to the formation of malondialdehyde. We have previously reported increases in lipid peroxidation with cells and found the iron content was responsible for the oxygen free radical-like damage (Turver *et al.*, 1985).

To investigate the role of free radicals in the action of asbestos, we have examined the production of malondialdehyde in cell cultures following treatment with asbestos and studied the effect of iron chelating agents on its production. A parallel study was also conducted to find whether asbestos had any effect on cell DNA strand breakage. Again scavengers of iron were added to test the hypothesis of an association between iron content and DNA-strand breakage.

Correspondence: R.C. Brown.
Received 9 January 1987.

### Materials and methods

### *Cell cultures*

The C3H10T½ cell line (Reznikoff *et al.*, 1973) was received from Dr W.J. Harris, Inveresk Research International, Musselburgh, Scotland, UK. Cells were cultured in Dulbecco's modification of Eagle's medium containing 10% heat inactivated foetal bovine serum, streptomycin ($50 \mu g ml^{-1}$), penicillin ($50 IU ml^{-1}$) and $3.6 g l^{-1}$ sodium bicarbonate. Cultures were incubated at 37°C in an atmosphere of 8% carbon dioxide in air. Tissue culture medium, serum reagents, and sterile plastic were obtained from Flow Laboratories, Irvine, Scotland and Gibco Europe, Paisley, Scotland, UK.

### *Chemicals*

6[$^3$H]-Thymidine specific activity 26 Ci mmol$^{-1}$ was supplied by Amersham International PLC, UK. Desferrioxamine mesylate was supplied by Ciba Laboratories, Horsham, UK. Other organic chemicals were from Sigma Chemical Company, Poole, Dorset, UK.

### *Dusts*

The UICC samples of asbestos (Timbrell & Rendall, 1971) were used, and a sample of the UICC crocidolite was ball milled for 8 h (Brown *et al.*, 1978) to provide a low cytotoxic dust. All dust samples were weighed and autoclaved dry and resuspended in culture medium by sonication immediately before addition to cell cultures.

### *Measurement of lipid peroxidation*

Lipid peroxidation was measured using the thiobarbituric acid (TBA) test for malondialdehyde using methods previously described (Gavino *et al.*, 1981). This method detects products of lipid free radical damage, particularly malondialdehyde, but also includes other lipid oxidation products and the term malondialdehyde was used synonymously with thiobarbituric acid reactive substances (TBARS). The experiments used cell cultures maintained in 56 $cm^2$ Petri dishes which were grown to confluence before treatment with dust for 24 h. The role of iron was studied using desferrioaxamine at a dose that was previously determined not to be toxic to the cells. A standard curve was constructed using known amounts of MDA generated by the acid hydrolysis of 1,1,3,3-tetramethoxypropane.

*DNA-strand breakage*

The method was based on that described by Collins *et al.* (1982) especially suitable for measuring dust induced DNA damage. C3H10T½ cells were inoculated into 8 chamber multislides (Lab-Tek division of Miles Laboratories) each chamber receiving $1 \times 10^4$ cells in 0.3 ml of medium. These cultures were then incubated for 2 days with 6-[$^3$H]-dT at 0.2 $\mu$Ci $ml^{-1}$ and grown to confluence. After this time they were washed and reincubated in non-radioactive medium. Two hours later this medium was replaced by medium containing 10 mM hydroxyurea and 10 $\mu$M 1-$\beta$-D-arabinosylfuranosylcytosine (AraC, Sigma Chemical Co.).

After 1 h suspensions of dust or a solution of 4-nitroquinoline-1-oxide as positive control was added, these solutions being made in medium containing the above inhibitors. After 24 h the chambers were removed from the slides and the monolayers washed in medium. In some experiments desferrioxamine was added with dust at a non-toxic dose and DMEM was replaced by MEM to reduce the effect of ferric iron present in the DMEM. The cells were then lysed in alkali by gently pipetting 50 $\mu$l of alkaline sucrose (5% w/v sucrose, 0.3 M NaOH, 0.5 M NaCl) onto each square. After 15 min at 4°C the alkaline cell lysate was brought to pH 4.5 with 15 $\mu$l of 2 M acetic acid. A 10 mm diameter nitrocellulose disc (BA85 Schleicher & Schuell) was placed over each square followed by a similarly sized GFB glass fibre disc (Whatman) to absorb the lysis solution. The plastic chamber unit previously removed from the slide was inverted and placed over the disc with a 25 g weight to exert even pressure during the transfer of DNA.

After 1 h at room temperature the chamber, weight and glassfibre disc were removed. Fifty $\mu$l of a solution containing 0.24 IU $ml^{-1}$ deoxyribonuclease-$S_1$ (Calbiochem) in sodium acetate buffer (0.03 M, pH 4.5 with $ZnSO_4$ at 30 $\mu$M) was added to each nitrocellulose disc. The slides were then incubated at 45°C for 45 min, a second glass fibre disc was then placed on each filter to absorb the supernatant. The nitrocellulose discs were treated with TCA, washed and dried. All the discs were then placed in scintillation vials with 2 ml of scintillant and counted. The percentage of radioactivity released from the nitrocellulose filter by the nuclease treatment is a measure of single-stranded DNA resulting from unwinding at the sites of damage.

### Results

*Effect of crocidolite asbestos on lipid*

The results in Figure 1 show that crocidolite asbestos is effective in stimulating the accumulation of TBARS in cultures of C3H10T½ cells. The effect of milling crocidolite is also shown and had no effect on the production of TBARS. Asbestos contains iron in its lattice structure which could participate in the production of TBARS. To investigate this hypothesis cultures of C3H10T½ cells were treated with asbestos which had been pretreated with desferrioxamine, a specific iron chelator. The results in Figure 1 show that desferrioxamine significantly reduced TBARS formation in both asbestos treated and control cultures. In the same experiment milled crocidolite was also effective in stimulating the accumulation of TBARS.

**Figure 1** Effect of desferrioxamine on the production of thiobarbituric acid reactive substances (TBARS) in C3H10T½ cultures treated with crocidolite (A) and milled crocidolite (B). Values are means with the s.d. indicated by an error bar derived from 5 cultures and expressed as the equivalent concentration of malondialdehyde (MDA). * indicates a significant difference at the 5% level; ** significantly different at the 1% level between desferrioxamine treated and untreated. (Student's *t*-test) □ no desferrioxamine mesylate; ▨ 300 $\mu$M desferrioxamine mesylate.

*Effect of crocidolite asbestos on DNA*

The alkaline strand separation method was used to detect the accumulation of DNA breaks produced by asbestos. The effect of fibre size and exposure time on DNA strand breakage is presented in Figure 2. When C3H10T½ cells were incubated with either milled crocidolite or the parent UICC crocidolite for only 2.5 h there was no significant increase in DNA-strand breakages as measured by the quantity of radioactive label released from the nitrocellulose-bound DNA following S1-nuclease treatment. In contrast, when these same concentrations were extended to 24 h all concentrations produced significant breakage compared with the control. Also, the ability of milled crocidolite to induce strand breakage indicates the phenomenon is largely independent of fibre morphology. The carcinogen 4-nitroquinoline-1-oxide was used as a positive control and induced DNA-strand breakages in a dose-dependent manner confirming the assay was responsive to chemically-induced DNA-damage.

The effect of desferrioxamine on strand breakage was investigated. Table I shows that pretreatment with desferrioxamine reduced asbestos DNA-strand breakage. The reduction was only small and the inhibition could have been affected by the presence of iron in the DMEM reducing the effectiveness of the desferrioxamine. To remove this possibility a second experiment was conducted in which the cells were treated in MEM which contains no added iron.

**Table I** The effect of desferrioxamine on crocidolite induced DNA-damage in C3H10T½ cells as measured by the $S_1$-nuclease digest method

| Treatment | | *% Release of nitrocellulose-bound DNA by $S_1$-Nuclease* | |
|---|---|---|---|
| | | *Desferrioxamine Mesylate* | |
| | $\mu$g $cm^{-2}$ | 0 | 300 $\mu$M |
| Control | | 34.04 ± 9.73(6) | 16.07 ± 4.21(6)$^b$ |
| Crocidolite | 200 | 63.20 ± 7.79(5)$^a$ | 51.61 ± 10.95(4)$^b$ |
| Milled Crocidolite | 200 | 70.45 ± 8.38(5)$^a$ | 52.37 ± 1.75(5) |

Values represent mean ± s.d. for the number of determinations in brackets. Analysis was by Student's *t* test. $^a$Indicates a significant difference from the control at the 5% level; $^b$Indicates a significant difference between desferrioxamine treated and untreated.

**Figure 2** The production of DNA-strand breaks in C3H10T½ cells treated with crocidolite and milled crocidolite. Twelve cultures in the control group were used and 6 in each treatment group. The mean values are shown with the s.d. indicated by an error bar. Results analysed by Student's *t*-test adjusting for multiple comparisons between groups, * indicates a significant difference between 2.5 h and 24 h exposure at the 5% level; □ no treatment; ▨ crocidolite 200 $\mu g\,cm^{-2}$; ▩ milled crocidolite 200 $\mu g\,cm^{-2}$; ▦ nitroquinoline N-oxide 2 $\mu g\,ml^{-1}$.

**Figure 3** Effect of desferrioxamine on the generation of DNA-strand breaks in C3H10T½ exposed to crocidolite. Twelve cultures in the control group were used and 6 in each dust treated group. The mean values are shown with the s.d. indicated by the error bar. * indicates a significant difference at the 5% level; ** significantly different at the 1% level between desferrioxamine treated and untreated. (Student's *t*-test); □ no desferrioxamine mesylate; ○ 300 μM desferrioxamine mesylate.

The results from this experiment are presented in Figure 3 and demonstrate that desferrioxamine produces significant inhibition of asbestos strand breakages.

### Discussion

The results above indicate crocidolite asbestos can induce lipid peroxidation and cause DNA damage in C3H10T½ cells by a mechanism involving iron. In previous studies asbestos has been shown to induce lipid peroxidation with red blood cells (Gabor & Anca, 1975), phospholipid emulsions (Weitzman & Weitberg, 1985) and isolated microsomes (Gulumian *et al.*, 1983). Others have shown the iron component of crocidolite fibres can catalyse the generation of hydroxyl radicals from hydrogen peroxide (Weitzman & Graceffa, 1984). We have previously shown that the lipid damaging activities of crocidolite is preventable by desferrioxamine treatment (Turver *et al.*, 1985). This has led to the suggestion that asbestos can cause cell toxicity by a modified Haber–Weiss reaction.

The generation of TBARS by crocidolite asbestos was found to be independent of fibre morphology. Milled (non-fibrous) crocidolite asbestos was as least as effective as the UICC sample, indicating a surface-related phenomenon. This effect is further supported again by our earlier observations and those of Weitzman and Graceffa (1984). The iron chelator desferrioxamine significantly reduced asbestos stimulated TBARS accumulation suggesting that the oxidative damage responsible for lipid peroxidation was produced by an iron-catalysed reaction. Amphibole asbestos, such as crocidolite, possesses significant amounts of ferrous and ferric iron linked into its crystal structure and has previously been shown to generate free radicals from hydrogen peroxide (in non-cellular systems). This type of mechanism has also been shown to damage isolated DNA when incubated with asbestos and hydrogen peroxide (Kasai & Nishimura, 1984).

A number of the toxic and pathogenic activities of asbestos fibres have been shown to be dependent on the size and shape of the dust (Brown *et al.*, 1978; Stanton *et al.*, 1977). Milled crocidolite is significantly less cytotoxic than parent dust towards C3H10T½ cells so the increased sensitivity of DNA from treated cells is not caused by an overall toxic phenomenon. The formation of strand breaks was also dependent on the length of incubation, there was little effect at 2.5 h while significant damage was seen after 24 h. The delay may represent the time for the dust to interact with the cells. In contrast 4-nitroquinoline-1-oxide produced detectable damage after both 2.5 h and 24 h incubation. Thus there are similarities between asbestos-induced lipid peroxidation and DNA-strand breaks which appear to be a product of surface chemistry rather than fibre morphology. Further insight into the DNA-damaging activity of asbestos was found from the effect of desferrioxamine on DNA-strand breakage. The addition of desferrioxamine to asbestos exposed cells caused a significant reduction in DNA-strand breakage. This was particularly evident when these experiments were carried out in medium containing little or no added iron.

The presence of increased DNA-strand breaks in asbestos-exposed cells would seem to support the idea that asbestos can cause direct genetic damage. The presence of this activity could also explain some earlier reports showing genotoxicity with asbestos (Huang *et al.*, 1978; Livingston *et al.*, 1980). However, there have been reports which have failed to detect DNA-strand breaks using alkaline elution in human cells (Fornace *et al.*, 1982). There may be a number of reasons for these variations: these authors used a different assay method; a shorter exposure time and with lower concentrations of dust. In this study milled crocidolite was found to be as active as the UICC crocidolite sample. In contrast, milled crocidolite has been found to be less tumourigenic in animals by intrapleural injection (Wagner *et al.*, 1984). It is possible that a combination of fibre size and chemistry may explain the added risks associated with crocidolite exposure.

In conclusion this study demonstrates that asbestos can cause DNA damage by a mechanism that depends on the presence of iron. The effect of desferrioxamine on strand breakage and lipid peroxidation is consistent with the concept that this damage is by a free-radical mechanism. However, these effects were not fibre-size dependent but may still represent an important mechanism in the aetiology of asbestos associated disease.

### References

BABU, K.A., NIGRAM, S.K., LAKKAD, B.C. & 5 others (1981). Effect of chrysolite asbestos (AP-1) on sister chromatid exchanges in Chinese hamster ovary cells. *Eviron. Res.*, **24**, 325.

BROWN, R.C., CHAMBERLAIN, M., GRIFFITHS, D.M. & TIMBRELL, V. (1978). The effect of fibre size on the *in vitro* biological activity of three types of amphibole asbestos. *Int. J. Cancer*, **22**, 721.

BROWN, R.C., POOLE, A. & FLEMING, G.T.A. (1983). The influence of asbestos dust on the oncogenic transformation of C3H10T$\frac{1}{2}$ cells. *Cancer Lett.*, **18**, 221.

CERUTTI, P.A. (1985). Prooxidant states and tumour promotion. *Science*, **227**, 375.

CHAMBERLAIN, M. & TARMY, E.M. (1977). Asbestos and glass fibres in bacterial mutation tests. *Mutat. Res.*, **43**, 159.

COLLINS, A., JONES, C. & WALDREN, C. (1982). A survey of DNA repair incision activities after ultraviolet irradiation of a human, hamster and human–hamster hybrid cell lines. *J. Cell. Sci.*, **56**, 423.

DOLL, N.J., STANKUS, R.P., GOLBACH, E. & SALVAGGIO, J.E. (1982). *In vitro* effects of asbestos fibres on polymorphonuclear leukocyte function. *Int. Arch. Allergy Appl. Immunol.*, **68**, 17.

EBERHARDT, M.K., ROMAN-FRANCO, A.A. & QUILES, M.R. (1985). Asbestos induced decomposition of hydrogen peroxide. *Environ. Res.*, **37**, 287.

FORNACE, A.J., LECHNER, J.F., GRAFSTROM, R.C. & HARRIS, C.C. (1982). DNA repair in bronchial epithelial cells. *Carcinogenesis*, **12**, 1373.

GABOR, S. & ANCA, Z. (1975). Effects of asbestos on lipid peroxidation in red cells. *Br. J. Indust. Med.*, **32**, 39.

GAVINO, V.C., IKAREBHA, S.O., MILO, G.E. & CORNWELL, D.G. (1981). Effects of polyunsaturated fatty acids on lipid peroxidation in cultures. *J. Lipid Res.*, **22**, 763.

GULUMIAN, M., SARDIANOS, F., KILROE-SMITH, T. & OCKERSE, G. (1983). Lipid peroxidation in microsomes induced by crocidolite fibres. *Chem. Biol. Interactions*, **44**, 111.

HESTERBURG, T.W. & BARRETT, J.C. (1984). Dependence of asbestos and mineral dust induced transformation of mammalian cells in culture on fibre dimension. *Cancer Res.*, **44**, 2170.

HUANG, S.L., SAGGIORO, D., MICHELMAN, H. & MALLING, H.V. (1978). Genetic effects of crocidolite asbestos in Chinese hamster lung cells. *Mutat. Res.*, **57**, 225.

HUANG, S.L. (1979). Amosite, chrysotile and crocidolite asbestos are mutagenic in Chinese hamster lung cells. *Mutat. Res.*, **68**, 265.

KASAI, H. & NISHIMURA, S. (1984). DNA damage induced by asbestos in the presence of hydrogen peroxide. *Gann*, **75**, 841.

LIVINGSTONE, G.K., ROM, W.N. & MORRIS, M.V. (1980). Asbestos induced sister chromatid exchanges in cultured Chinese hamster ovarian fibroblast cells. *J. Environ. Pathol. Toxicol.*, **4**, 373.

MOSSMAN, B.T. & LANDESMAN, J.M. (1983). Importance of oxygen-free radicals in asbestos-induced injury to airway epithelial cells. *Chest*, **83**, 505.

PRICE-JONES, M.J., GUBBINGS, G. & CHAMBERLAIN, M. (1980). The genetic effects of crocidolite asbestos. Comparison of chromosome abnormalities and sister chromatid exchanges. *Mutat. Res.*, **79**, 331.

REISS, B., SOLOMON, S., TONG, C. & 3 others (1982). Absence of mutagenic activity of three forms of asbestos in liver epithelial cells. *Environ. Res.*, **27**, 389.

REZNIKOFF, C.A., BRANKOW, D.W. & HEIDELBERGER, C. (1973). Establishment and characterisation of a cloned line of C3H mouse embryo cells sensitive to post confluence inhibition of division. *Cancer Res.*, **33**, 3231.

SELIKOFF, I.J. & LEE, D.H.K. (1980). *Asbestos and Disease*, Academic Press, New York.

SINCOCK, A. & SEABRIGHT, M. (1975). Induction of chromosal changes in Chinese hamster cells by exposure to asbestos dusts. *Nature*, **258**, 56.

STANTON, M.F., LAYARD, M., TEGERIS, A. & 3 others (1977). Carcinogenicity of fibrous glass: pleural response to fibre dimension. *J. Natl Cancer Inst.*, **58**, 797.

TIMBRELL, V. & RENDALL, R.E.G. (1971). Preparation of the UICC standard reference samples of asbestos. *Powder Technol.*, **5**, 279.

TURVER, C.J., BROWN, R.C. & POOLE, A. (1985). Lipid peroxidation and the generation of malondialdehyde in asbestos treated cell cultures. In *The In Vitro Effects of Mineral Dusts*, (eds) Beck, E.G. & Bignon, J. p. 267. NATO ASI Series G-3. Springer Verlag, Berlin.

WAGNER, J.C., SLEGGS, C.A. & MARCHAND, P. (1960). Diffuse pleural mesothelioma and asbestos exposure in the North Western Cape Province. *Br. J. Ind. Med.*, **17**, 260.

WAGNER, J.C., BERRY, G. & TIMBRELL, V. (1973). Mesotheliomata in rats after inoculation with asbestos and other minerals. *Br. J. Cancer*, **28**, 173–185.

WAGNER, J.C., GRIFFITHS, D.M. & HILL, R.J. (1984). The effect of fibre size on the *in vivo* activity of UICC crocidolite. *Br. J. Cancer*, **49**, 453.

WEITZMANN, S.A. & GRACEFFA, P. (1984). Asbestos catalyses hydroxyl and superoxide radical generation from hydrogen peroxide. *Arch. Biochem. Biophys.*, **228**, 373.

WEITZMAN, S.A. & WEITBERG, A.B. (1985). Asbestos catalysed lipid peroxidation and its inhibition by desferrioxamine. *Biochem. J.*, **225**, 259.